# FACS-based isolation and recovery of physiologically distinct bacterial subpopulations from infected insect hosts

**DOI:** 10.1016/j.mex.2026.104194

**Published:** 2026-10-07

**Authors:** Hasna Toukabri, Christophe Buisson, Yves Le Vern, Alix Sausset, Mickaël Bourge, Leyla Slamti

**Affiliations:** a Micalis Institute, INRAE, AgroParisTech, Université Paris-Saclay, Jouy-en-Josas, 78350, France; b Unité Mixte de Recherches (UMR) 1282 Infectiologie et Santé Publique, INRAE Centre Val de Loire, Université François Rabelais de Tours, Nouzilly, France; c Université Paris-Saclay, CEA, CNRS, Institute for Integrative Biology of the Cell (I2BC), Gif-sur-Yvette, France

**Keywords:** FACS, *Bacillus thuringiensis*, *Galleria mellonella*, Infection, Phenotypic heterogeneity, Sporulation, Bacterial adaptation, Persistence, Virulence, Bacterial sorting

## Abstract

Phenotypic heterogeneity is often encountered during infection but remains challenging to investigate *in vivo*. Here, using the entomopathogenic spore-former *Bacillus thuringiensis* with the *Galleria mellonella* insect larva infection model and Fluorescence-Activated Cell Sorting (FACS), we present a workflow that enables the isolation of bacterial subpopulations in distinct physiological states, directly from infected hosts, for downstream characterization. Host-derived debris and the low bacterial recovery after sorting represent major limitations of *in vivo*-derived FACS approaches. By combining pre-sorting filtration, optimized FACS parameters, and post-sorting processing, the workflow minimizes host-derived contamination while preserving bacterial viability and physiological integrity. The recovered subpopulations are suitable for a wide range of downstream characterization, including reinfection experiments, tolerance assays, infection cycle studies and competition assays. As an illustrative application, we used this method on larvae infected with a strain harboring a fluorescent reporter that marks cells irreversibly committed to sporulation. Sporulating and non-sporulating bacteria recovered from the insects were successfully separated and subsequently used in reinfection experiments.

**• Isolation of physiologically distinct bacterial subpopulations directly from infected insect hosts**

**• FACS-based separation of bacterial subpopulations using fluorescent reporters**

**• Recovery of viable sorted bacterial populations for downstream characterization**

## Graphical abstract

**Figure:**
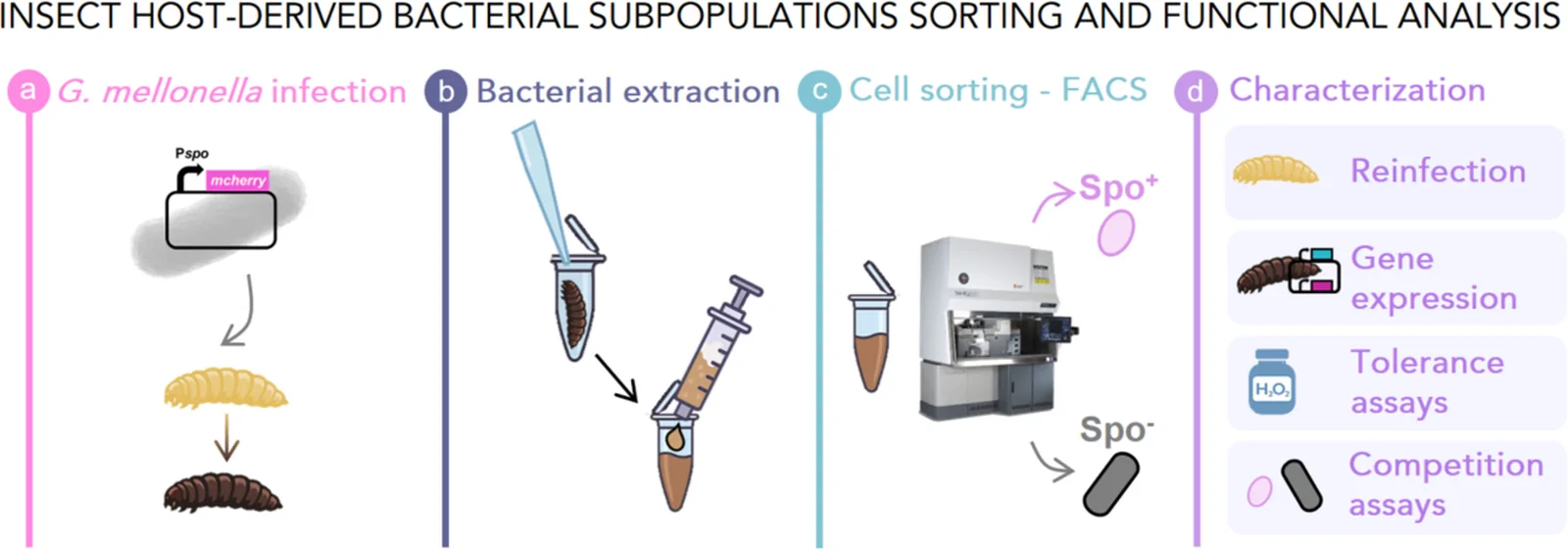

## Specifications table

**Table:** 

**Subject area**	Immunology and Microbiology
**More specific subject area**	Host-pathogen interaction and flow cytometry
**Name of your method**	FACS-based isolation and recovery of physiologically distinct bacterial subpopulations from infected insect hosts
**Name and reference of original method**	None
**Resource availability**	**Biological material***G. mellonella* larvae, third instar, 60 mg ± 10 mg — (in-house rearing in this study, but larvae can be purchased)**Bacterial strains** • *B. thuringiensis* strain Bt (pP*spoIIQ*’*mcherry*) (*B. thuringiensis* strain 407^−^ in which we measure the activity of the promoter of *spoIIQ*, using an mCherry-encoding reporter gene [1]) — stored at -80 °C in LB + 15% glycerol. **Reagents** • Sterile saline (0.9% NaCl) • PBS (137 mM NaCl Supelco cat.no.1.06104.1000, 2.7 mM KCl Sigma-Aldrich, cat.no. P3911, 8 mM Na2HPO4 cat.no.PRO-28028.298, 2 mM KH2PO4 Merck cat.no.1.06579.0500) • LB broth — BD, cat. no. 244620 • LB agar • Agar — Invitrogen, cat. no. 30391023 **Equipment** • Microinjector / syringe pump — e.g., KD Scientific Legacy Syringe Pump, Sigma-Aldrich, cat.no. Z401366 • Syringes without needle, luer slip tip, 1 mL — Terumo, cat.no.MDSS01SE • Syringes without needle, luer slip tip, 2,5 mL — Terumo, cat.no.SS*02SE1 • Carded cotton — Dutscher, cat.no.351400 • Needles 0.45 × 13 mm — Terumo, cat.no.AN*2613R1. • Camera — Nikon CoolPix P1 • 6- or 24-well cell culture plates — Sarstedt, cat.no.83.3920 (6 wells) and 83.3922 (24 wells) • Entomological forceps • Bead-beater-adapted tubes (2 mL, flat-bottom) — Sarstedt, cat.no. 72.694 • 3 mm sterile glass beads — Sigma-Aldrich, cat.no.Z143928 • Bead-beater homogenizer — FastPrep MP Biomedicals FastPrep-24TM Classic, cat.no.116004500 • 0.22 µm sterile filter units (Steritop or equivalent) — Millipore, cat.no.S2GPU01RE • Micro-centrifuge — Eppendorf MiniSpin, cat.no.5452000010 • Thermostatic dry bath — ThermoStat C Eppendorf, cat.no.5383000019 • 1,5 mL tubes • 15 mL tubes • 50 mL tubes • Flow cytometer — CyFlow Space, Sysmex Partec, France (analysis) • Cell sorter — MoFlo AstriosEQ, Beckman Coulter (sorting) • Glass vacuum filtration system — Merck cat.no.XX1012500 • 0.22 µm PES membrane filter — Millipore cat.no. GPWP09050 • Filter forceps — Merck cat.no. XX6200006P • Micropipettes • Multipipettes • Micropipet tips • Incubator/shaker **Softwares** • FloMax • Summit • Kaluza • Weasel • GraphPad Prism

## Background

Isolation of bacterial subpopulations directly from infected hosts is essential for understanding population heterogeneity during infection, yet it remains technically challenging in host–microbe systems. Recent studies have demonstrated that fluorescent reporters combined with single-cell approaches reveal bacterial states involved in stress adaptation, differentiation and persistence that cannot be resolved using conventional microbiological assays [[1], [2], [3]]. Using fluorescent reporters, fluorescence-activated cell sorting (FACS) enables the identification and isolation of bacterial subpopulations displaying distinct physiological states, allowing the analysis of phenotypic heterogeneity that is masked in bulk population analyses [[4], [5], [6]]. However, applying FACS to host-derived bacterial samples remains technically challenging. Host debris increase background signals and complicate accurate bacterial discrimination and gating prior to sorting [7]. In addition, sorted fractions are typically recovered in large volumes with limited bacterial recovery, restricting their direct use in downstream functional assays. While FACS has been successfully applied for phenotypic profiling and molecular characterization of sorted bacterial states, including DNA- and RNA-based analyses [8,9], the recovery of sufficient numbers of viable cells from host for reinfection, stress-response or fitness assays remains challenging.

To address this gap, we developed and optimized a bacterial isolation and collection procedure that enables the recovery of viable bacterial subpopulations directly from infected insect hosts while minimizing host-derived contamination. The procedure combines syringe-based filtration to remove insect-derived material, and membrane-based recovery to reduce the final sample volume while retaining viable bacteria for FACS sorting and downstream functional assays. We used the *Bacillus thuringiensis–Galleria mellonella* infection model. *G. mellonella* is an increasingly used ethical model for infection studies, combining a conserved innate immune response with experimental accessibility, while allowing recovery of sufficient bacterial biomass for bacterial physiological analysis [[10], [11], [12]]. During *G. mellonella* infection, *B. thuringiensis* completes a quorum-sensing-regulated cycle involving virulence, necrotrophism, and sporulation [13]. Single-cell studies of the infection process revealed heterogeneous populations of sporulating and persistent non-sporulating cells with slowed-down metabolism and increased oxidative stress resistance [1,2]. However, how host passage affects the bacterial physiology and the dynamics of these subpopulations remained unclear. We therefore developed a workflow to isolate sporulating (Spo⁺) and non-sporulating (Spo⁻) *B. thuringiensis* subpopulations from *G. mellonella* larvae. The recovery of viable sorted bacteria allowed downstream assays, including reinfection and competition experiments.

The workflow presented here combines host-derived debris removal with FACS-based separation of fluorescently labelled physiological states, enabling the recovery of intact bacterial subpopulations in reduced final volumes. Minimizing the time between host disruption, sorting, and recovery limits perturbations associated with prolonged handling and helps maintain the physiological state of sorted populations. Bacterial recovery from infected larvae is commonly performed using dedicated homogenization equipment such as an Ultra-Turrax or FastPrep [[14], [15], [16]]. In contrast, our workflow uses a simple 1-mL tip for homogenization followed by filtration with a syringe loaded with carded cotton, requiring neither dedicated equipment nor extensive handling. This rapid, low-equipment approach facilitates direct processing of samples. In parallel, the use of simple aqueous buffers commonly employed for physiological analyses ensures compatibility with live-cell processing. These combined features improve the quality, viability, and consistency of recovered bacterial fractions. By maintaining bacterial integrity throughout the workflow, this approach provides a robust framework for studying host-driven phenotypic heterogeneity at the subpopulation level (Graphical abstract and Fig. 1).

**Fig. 1:**
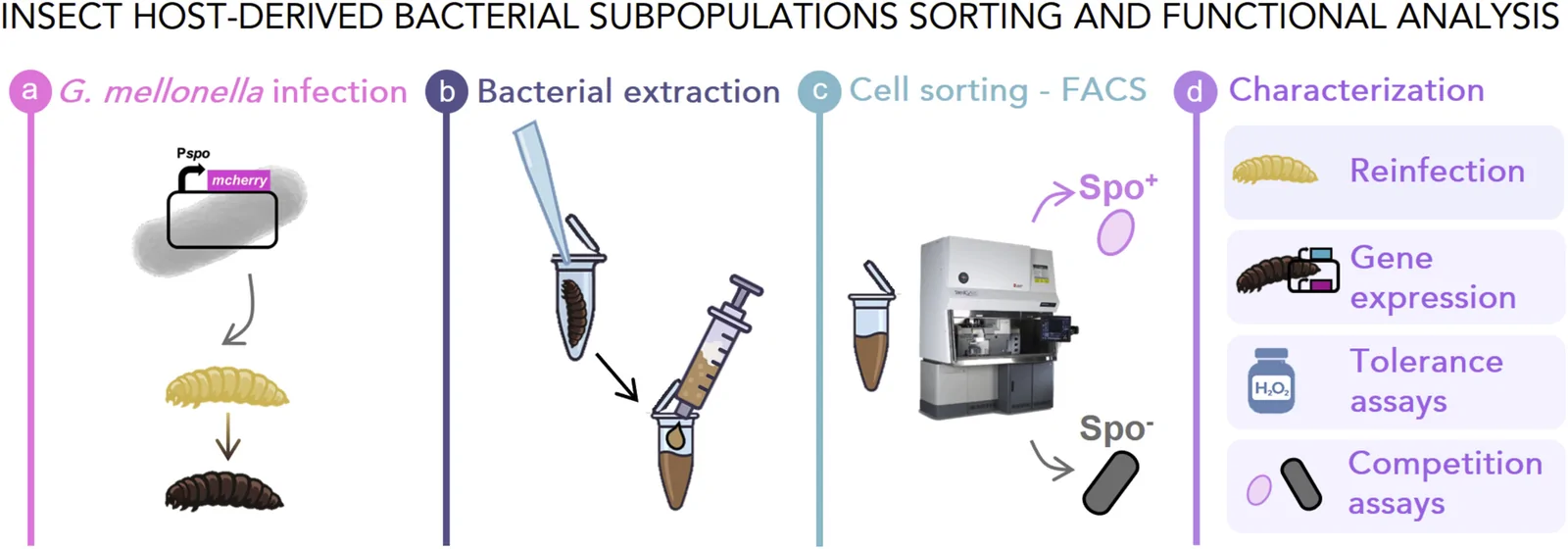
**Overview of the workflow for isolation and characterization of insect host-derived bacterial subpopulations. (a) Infection of *G. mellonella* larvae.** Third-instar larvae (60 ± 10 mg) are infected with *B. thuringiensis* strain Bt (p*PspoIIQ’mcherry*) carrying a sporulation reporter to identify Spo^+^ (mCherry-positive) and Spo^−^ (mCherry-negative) subpopulations. **(b) Bacterial recovery from infected hosts.** Infected larvae cadavers are homogenized and filtered through a carded cotton-plugged syringe to remove insect debris and obtain clean bacterial suspensions suitable for sorting. **(c) FACS.** Bacterial populations are separated according to mCherry fluorescence, allowing isolation of Spo⁺ (mCherry-positive) and Spo⁻ (mCherry-negative) subpopulations. **(d) Characterization.** Sorted bacterial subpopulations are recovered and used for downstream assays, including reinfection, stress tolerance, gene expression, and competition analyses.

## Method details

### Sample preparation for FACS application

#### Extraction of bacterial cells from infected *G. mellonella* larvae

*G. mellonella* larvae (third instar, 60 ± 10 mg) were infected by intrahemocoelic injection with *B. thuringiensis* strain Bt (pP*spoIIQ*’*mcherry*) to detect and characterize sporulating (Spo^+^, red fluorescent) and non-sporulating (Spo^−^, non-fluorescent) subpopulations. An appropriate fluorescent reporter or labeling strategy can be used to specifically identify the microorganism or physiological subpopulation of interest within a complex host-derived sample, including in the presence of the host microbiota. Therefore, the workflow is compatible with alternative fluorescent reporter combinations depending on the biological question. Following infection, bacterial suspensions were prepared using a standardized recovery procedure. Infected *G. mellonella* larvae were processed at the desired infection time point. Larvae were individually transferred into 1.5 mL microcentrifuge tubes, crushed using a 1 mL pipette tip and 1 mL sterile saline (0.9% NaCl) was added. Homogenates were centrifuged for 45 s at 12,100 × g. The bacterial pellet was resuspended in 750 µL sterile saline and filtered through carded cotton placed into a 2.5 mL syringe to remove large insect debris. 1 µL of this suspension in 1 mL saline was then used for the sorting procedure (Fig. 2A).

**Fig. 2:**
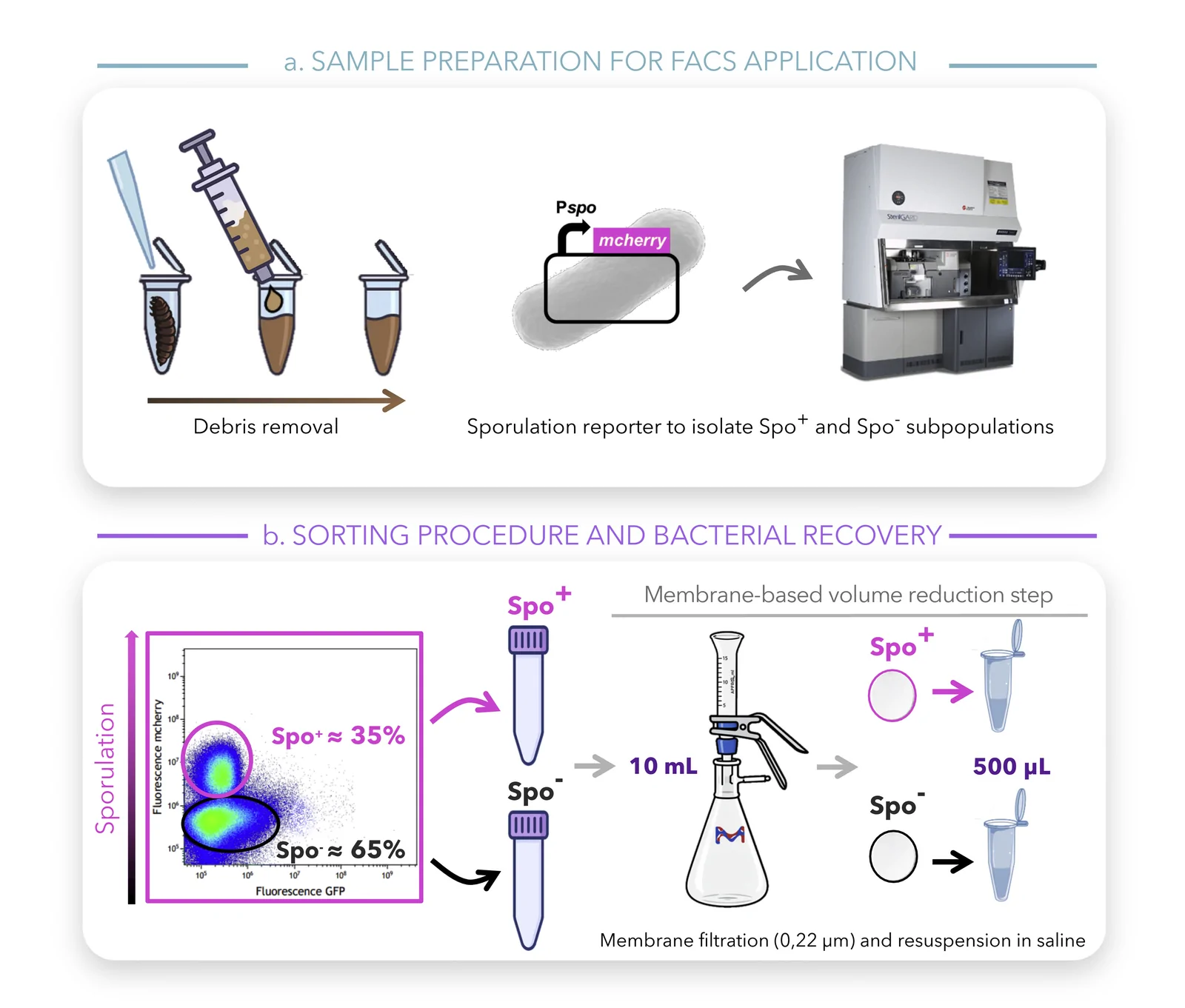
**FACS-based isolation and membrane-filtration recovery of bacterial subpopulations from infected insect hosts. (a) Sample preparation for FACS application.***B. thuringiensis* cells recovered from infected *G. mellonella* larvae were processed for flow cytometry analysis. Larvae were mechanically homogenized, and the resulting bacterial suspensions were filtered through a carded cotton-plugged syringe to remove host-derived debris prior to FACS acquisition*.* Bacterial cells carrying the sporulation reporter P*spoIIQ*’*mcherry* were analyzed based on their fluorescence profile, allowing discrimination of Spo^+^ (mCherry-positive) and Spo^−^ (mCherry-negative) subpopulations. **(b) Sorting procedure and bacterial recovery.** FACS gating based on fluorescence intensity enabled separation of Spo⁺ (mCherry-positive, pink) and Spo⁻ (mCherry-negative, black**)** bacterial subpopulations. 10 mL of sorted fractions were collected independently into sterile tubes and subsequently collected on a 0.22 µm membrane using a filtration unit connected to a vacuum system. Recovered bacterial suspensions were resuspended in 500 µL of saline for downstream analyses. This workflow allows the isolation of physiologically distinct bacterial subpopulations from complex host-derived samples while preserving bacterial integrity during and after sorting.

**Critical step:** Processing time during bacterial extraction from insect cadavers should be minimized to reduce post-mortem changes in bacterial physiology and aggregation of host debris. A second filtration step can be performed to remove residual debris.

### Sorting procedure and bacterial recovery

#### Flow cytometric identification and sorting of bacterial subpopulations

Bacterial Spo^+^ and Spo^−^ subpopulations were sorted using a MoFlo AstriosEQ cell sorter (Beckman-coulter). A 70-µm nozzle was used with a 60 PSI sheath pressure. To avoid bactericides or NaN3 found in commercial sheath liquid, home-made PBS was used (137 mM NaCl Supelco cat.no.1.06104.1000, 2.7 mM KCl Sigma-Aldrich, cat.no. P3911, 8 mM Na2HPO4 cat.no.PRO-28028.298, 2 mM KH2PO4 Merck cat.no.1.06579.0500). Sorter calibration was performed with Ultra Rainbow Calibration particles (Spherotech). Forward and side scatter (FSC/SSC) parameters were used to define the bacterial population and discriminate cells from background particles. For fluorescence detection, mCherry signal was collected using a 620/29-nm bandpass filter after excitation with a solid yellow/green laser emitting at 561 nm. Events rate was typically maintained at ∼40,000 per second, with a drop formation frequency close to 96,000 Hz.

### Gating strategy

A sequential gating strategy was applied (Fig. 3):

a. **FSC-Height vs SSC-Height gate** to define the bacterial population and exclude residual insect debris.
b. **Doublet discrimination** using SSC-Area vs SSC-Height plots.
c. **Fluorescence gating** to separate subpopulations: Spo⁺ (red fluorescent, mCherry-positive cells) and Spo⁻ (non-fluorescent, mCherry-negative cells)

**Fig. 3:**
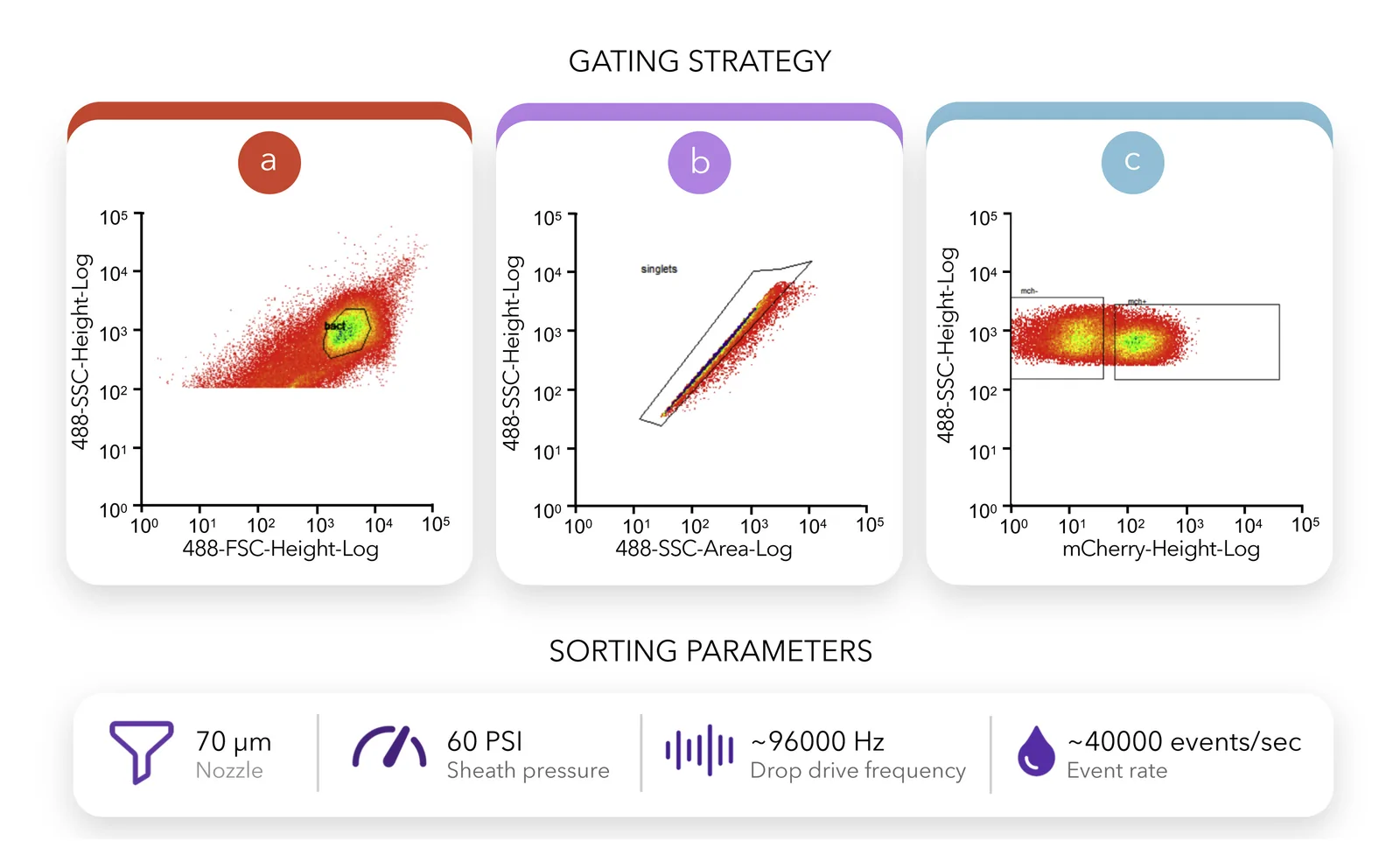
**FACS gating strategy for isolation of sporulating and non-sporulating bacterial subpopulations from infected insect hosts.** A sequential gating strategy was applied to isolate *B. thuringiensis* subpopulations recovered from infected *G. mellonella* larvae. (a) Bacterial events were selected based on FSC-Height and SSC-Height parameters to exclude residual host-derived debris. (b) Doublets were excluded using SSC-Area versus SSC-Height discrimination. (c) Fluorescence-based gating using the P*spoIIQ*’*mcherry* reporter allowed separation of Spo⁺ (mCherry-positive) and Spo⁻ (mCherry-negative) populations. Sorting was performed using a MoFlo AstriosEQ cell sorter equipped with a 70 µm nozzle at 60 PSI sheath pressure. Sorting conditions were maintained at approximately 40,000 events/s with a drop formation frequency of ∼96,000 Hz.

**Critical step:** High amounts of insect-derived debris require cotton filtration, a 100-fold dilution, and stringent gating to minimize contamination of sorted bacterial fractions.

#### Post-sorting recovery and preparation of bacterial samples

FACS-sorted bacterial populations can be directly used from the original collection volume for downstream analyses. Some applications require defined low-volume inocula, such as standardized 10 µL injections for *G. mellonella* reinfection experiments [17,18]. For these applications, sorted bacteria can be subjected to a vacuum-assisted membrane-based volume reduction procedure. This procedure decreases the sample volume from 10 mL to 500 µL while recovering viable bacteria (Fig. 2B).

For each condition, the sorter was programmed to collect 1 × 10⁷ bacterial events. Viable bacterial recovery after sorting was assessed by plating serial dilutions on LB agar. Approximately 8.2 × 10⁶ CFU were recovered from the 10 mL sorted suspension (8.2 × 10⁵ CFU/mL), corresponding to an overall recovery efficiency of ∼82%. When a reduced-volume format was preferred, samples were processed using a glass vacuum filtration system equipped with a sterile 0.22 µm PES membrane filter (Millipore). While membrane-based processing may influence overall bacterial recovery, it generated viable low-volume bacterial suspensions suitable for downstream functional assays, including *G. mellonella* reinfection experiments. This reduced-volume format facilitates handling of sorted fractions initially recovered in large collection volumes. The procedure is as follow:

1. The sorted bacterial suspension was gently loaded onto the filtration unit containing the 0.22 µm PES membrane.
2. Vacuum was applied until complete passage of the liquid phase, allowing bacterial cells to be retained on the membrane surface.
3. The membrane was carefully transferred into a sterile 1.5 mL microcentrifuge tube using sterile filter forceps.
4. Bacterial cells were recovered by gently washing the membrane surface with **500 µL sterile saline** followed by gentle vortexing.

**Critical step:** The membrane transfer step must be quick to avoid drying before resuspension.

### Downstream assays

Recovered bacterial subpopulations can be used in different representative downstream applications demonstrating the versatility of the workflow. For reinfection assays, sorted populations were adjusted to equivalent viable counts and injected into third-instar *G. mellonella* larvae in an injection volume of 10 µL containing 2 × 10³ CFU. Furthermore, independently sorted fluorescently labelled subpopulations were mixed at defined proportions to perform *in vitro* and *in vivo* competition assays, with their relative abundance monitored by flow cytometry after recovery. Additional functional or phenotypic assays can be performed using the recovered bacterial fractions according to the experimental objectives.

**Critical point:** Downstream assays should be initiated as rapidly as possible after collection to preserve physiological states acquired *in vivo*.

## Method validation

### *Validation of bacterial viability during sample handling*

To evaluate whether sample handling during the sorting procedure affected bacterial viability, colony-forming units (CFU/mL) were quantified after incubation of bacterial suspensions in sterile saline and sterile PBS, the two buffers used in the workflow. We collected bacteria from *G. mellonella* cadavers 7 days post-infection based on our previous publication [1] and incubated them in each buffer for 0, 1, 2 or 3 h, the latter time point corresponding to one hour after the maximum duration of sample preparation, FACS processing and bacterial recovery. Across conditions, CFU counts remained largely stable during the first hours of incubation, with only a limited decrease (approximately 10–15%) at 3 h (Fig. 4A). No marked difference was observed between saline and PBS. These results indicate that the buffers and processing times used in the workflow preserve bacterial cultivability. Consequently, processing samples within 3 h minimizes loss in viable bacteria and provides reproducible starting material for downstream experiments.

**Fig. 4:**
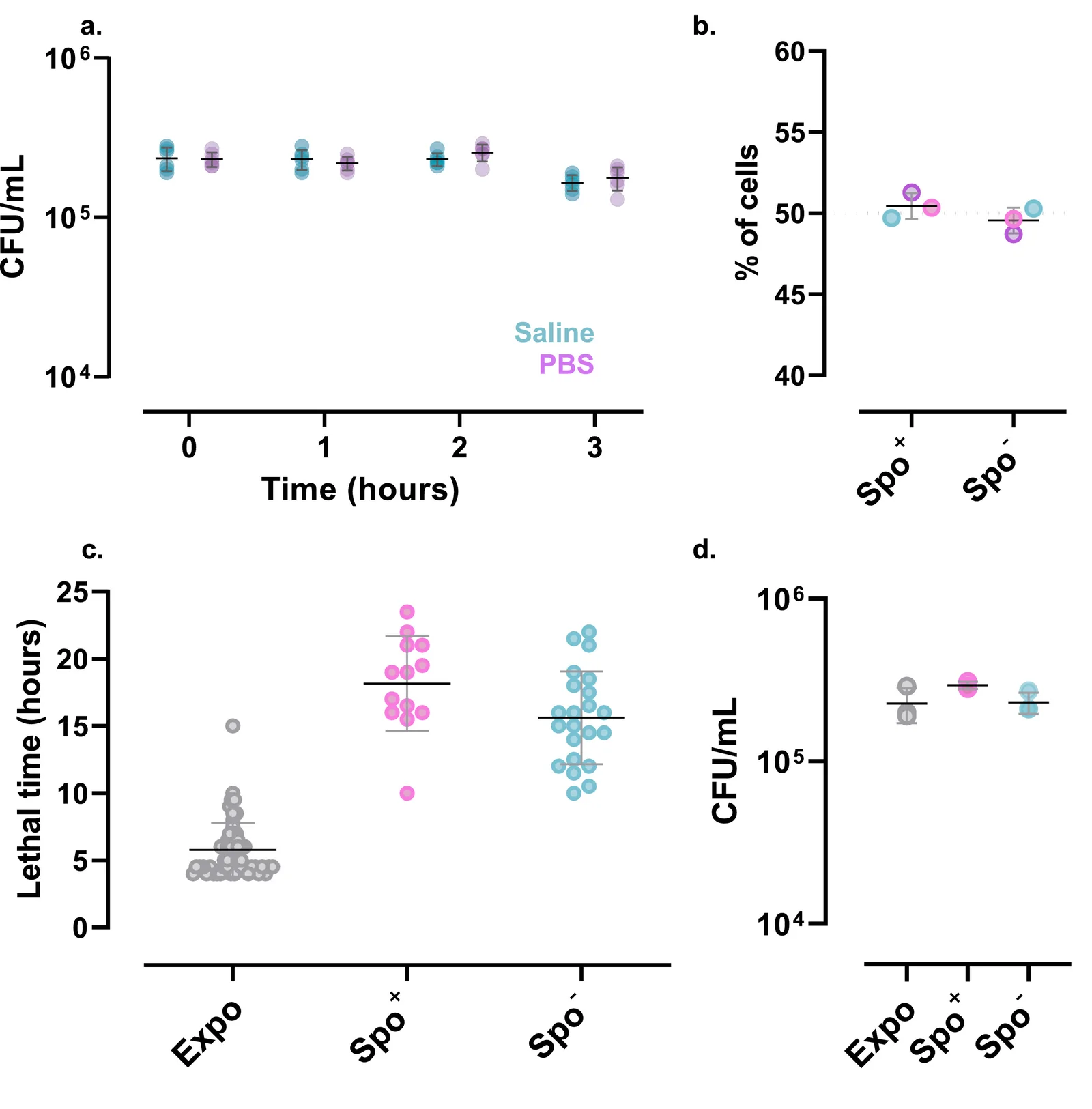
**Validation of the FACS-based isolation and recovery workflow for bacterial subpopulations from infected insect hosts. (a) Preservation of bacterial viability during sample handling.** Bacterial suspensions recovered from *G. mellonella* larvae infected with *B. thuringiensis* strain Bt (pP*spoIIQ*’*mcherry*) were incubated in sterile saline (blue) or PBS (purple) for 0, 1, 2 and 3 h to evaluate the impact of sample handling duration on bacterial viability. Colony-forming units (CFU/mL) were determined by serial dilution and plating on LB agar. Each symbol represents an independent biological replicate from one larva. **(b) Validation of controlled mixing of sorted bacterial subpopulations.** Spo⁺ (mCherry⁺) and Spo⁻ (mCherry⁻) subpopulations were independently sorted from infected larvae at 7 days post-infection, mixed at a 1:1 ratio, and analyzed by flow cytometry to determine the relative proportion of each population after mixing. The percentage of Spo⁺ and Spo⁻ cells is represented for three independent biological replicates. Identical colors correspond to the same biological replicate. **(c) Functional validation of sorted bacterial subpopulations through reinfection assays.** Membrane-collected sorted bacterial populations were used to prepare standardized inocula containing 2 × 10³ CFU in 10 µL for intrahemocoelic injection into third-instar *G. mellonella* larvae. *In vitro*-grown exponential-phase cells (gray) and Spo⁺ (pink) and Spo⁻ (blue) subpopulations recovered from infected larvae at 7 days post-infection were used for reinfection experiments. Individual lethal times recorded during the first 24 h post-infection are represented. At least 13 larvae per condition were analyzed. **(d) Quantification of bacterial inocula used for reinfection assays.** The bacterial concentration of each inoculum described in panel c was determined by CFU enumeration before injection. CFU/mL values are shown for exponential-phase cells (gray) and Spo⁺ (pink) and Spo⁻ (blue) sorted subpopulations. Three independent experiments were performed. Each symbol represents an independent biological replicate. Horizontal black bars indicate the mean, and error bars represent the standard deviation (SD).

### *Validation of mixed sorted bacterial subpopulations*

To assess whether sorted bacterial subpopulations could be accurately combined for downstream competitions assays, Spo⁺ (mCherry⁺) and Spo⁻ (mCherry^−^) subpopulations from infected larvae were independently sorted and mixed at an expected 1:1 ratio. The resulting mixtures were then analyzed by flow cytometry to determine the relative proportion of each subpopulation. Across three independent biological replicates, the relative proportions of mCherry⁺ and mCherry^−^ cells remained close to the expected 50:50 distribution, with paired Spo⁺ and Spo⁻ populations from each replicate showing similar values (Fig. 4B). These results indicate that the sorting and recovery workflow enables reproducible preparation of defined bacterial mixtures without introducing major biases toward one physiological state. This validation demonstrates that sorted bacterial populations can be recovered and recombined in controlled proportions, providing a suitable basis for downstream assays requiring direct comparison or interaction between distinct physiological states.

### Validation of bacterial reinfection assays following sorting and recovery

To validate that sorted bacterial populations remained suitable for downstream reinfection assays, membrane-collected suspensions were used to prepare 10 µL inocula containing 2 × 10³ CFU for *G. mellonella* reinfection. *In vitro*-grown exponential-phase bacteria and Spo⁺ and Spo⁻ subpopulations recovered from infected larvae at 7 days post-infection were compared under identical inoculation conditions. The resulting mortality, represented as individual larval lethal times during the first 24 h, showed distinct profiles among the three bacterial populations, demonstrating that the workflow preserves biologically relevant phenotypic differences following sorting and recovery (Fig. 4C). CFU-enumeration of the inocula confirmed that equivalent bacterial doses were administered for all experimental conditions (Fig. 4D). These results demonstrate that the workflow enables recovery of viable bacterial subpopulations in sufficient quantity and good physiological condition to perform reproducible reinfection assays using defined bacterial inputs (Fig. 4).

## Limitations

The workflow was developed and validated using the *B. thuringiensis–G. mellonella* infection model. Its applicability to other host–microorganism systems remains to be established and may require substantial adaptation depending on host-derived material, bacterial size and bacterial population size, aggregation properties, and susceptibility to mechanical processing. In addition, FACS-based identification and recovery requires a suitable fluorescent reporter or labeling strategy to distinguish the bacterial population or physiological subpopulations of interest. Thus, while the workflow provides a practical approach for recovering physiologically distinct bacterial subpopulations from infected hosts, its performance and applicability should be evaluated on a case-by-case basis in other biological systems. Finally, while the membrane-based volume reduction step provides sufficient viable bacteria for downstream assays, it may not recover the entire sorted population and is therefore particularly suited for applications requiring reduced working volumes.

## Ethics statements

All experiments involving invertebrates were conducted in accordance with the national guidelines for the ethical use of invertebrates in research.

## CRediT author statement

Conceptualization: H.T. and L.S.; Methodology: H.T.; Y.L.; M.B.; L.S.; Investigation: H.T.; C.B.; Y.L.; A.S.; M.B.; Visualization: H.T.; Funding acquisition: L.S. M.B.; Project administration: L.S.; Supervision: L.S.; Writing – original draft: H.T.; Writing – review and editing: H.T.; C.B.; Y.L.; A.S.; M.B.; L.S.

## Declaration of competing interest

The authors declare that they have no known competing financial interests or personal relationships that could have appeared to influence the work reported in this paper.

## Data Availability

Data will be made available on request.
